# (3^1^ *E*,3^2^ *Z*,7^1^ *E*,7^2^ *Z*)-4,8-Bis(3,5-di­chloro­phen­yl)-1^4^,3^3^,5^3^,7^3^-tetra­propyl-1^1^ *H*,3^2^ *H*,5^1^ *H*,7^2^ *H*-1,5(2,5),3,7(5,2)-tetra­pyrrola-2,6(2,5)-di­thiophena­cyclo­octa­phane

**DOI:** 10.1107/S2414314623007666

**Published:** 2023-09-08

**Authors:** Yoshihiro Ishimaru, Ryo Ikeda, Takashi Fujihara

**Affiliations:** a Division of Material Science, Graduate School of Science and Engineering, Saitama University, Shimo-ohkubo 255, Sakura-ku, Saitama City, Saitama, 338-8570, Japan; b Comprehensive Analysis Center for Science, Saitama University, Shimo-ohkubo 255, Sakura-ku, Saitama City, Saitama 338-8570, Japan; Zhejiang University (Yuquan Campus), China

**Keywords:** crystal structure, anti­aromatic, heteroatom, macrocyclic compound

## Abstract

Purple crystals of the title compound, C_50_H_44_Cl_4_N_4_S_2_ were obtained from the reaction of 2,5-bis­(4-propyl-1*H*-pyrrol-2-yl)thio­phene and 3,5-di­chloro­benzaldehyde in the presence of tri­fluoro­acetic acid for 3 h and subsequent addition of *p*-chloranil. The macrocycle in the title compound can be described as a highly planar structure wthe the average deviation of the 32 macrocyclic atoms from the least-squares plane being 0.0416 Å. Its mol­ecular conformation is stabilized by two intra­molecular N—H⋯N bonds and a three-dimensional network is formed by C—H⋯π inter­actions.

## Abstract 2

In the title compound, [24]di­thia­amethyrin(1.0.0.1.0.0); C_50_H_44_Cl_4_N_4_S_2_, the macrocycle can be described as a highly planar structure with a mean-plane deviation (MPD) value of 0.0416 Å. The mol­ecular conformation is stabilized by two intra­molecular N—H⋯N inter­actions. In the crystal, mol­ecules are also linked *via* C—H⋯π inter­actions forming a three-dimensional network.

## Graphical abstract

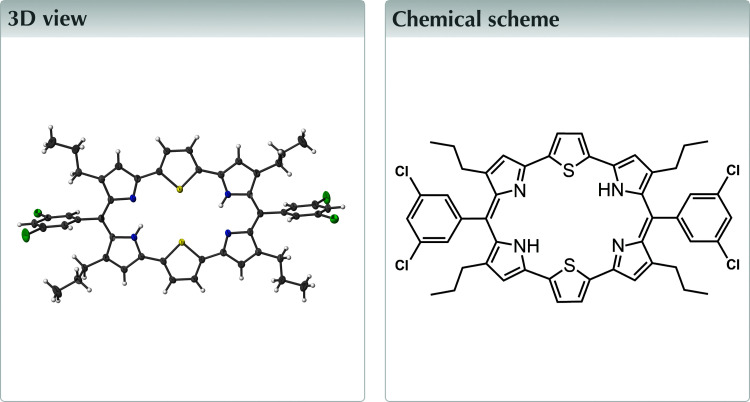

## Structure description

**Figure 1:**
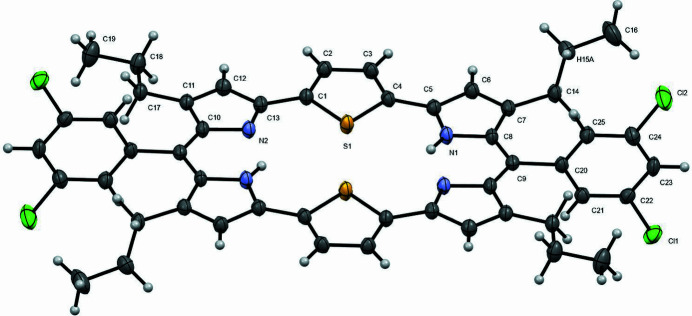
The mol­ecular structure of the title compound showing the atom labelling. Displacement ellipsoids are drawn at the 50% probability level. Non-labeled atoms are generated by symmetry operation −*x* + 1, −*y* + 1, −*z* + 1.

**Figure 2:**
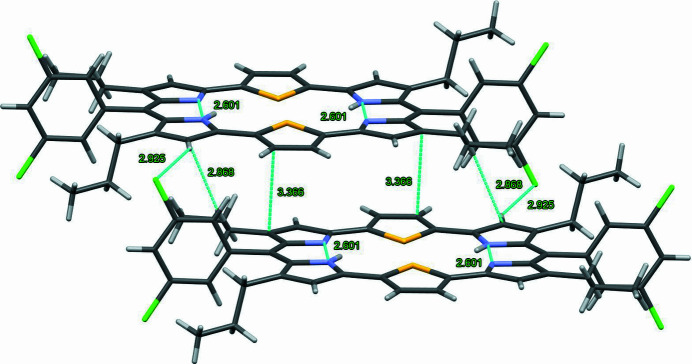
View of the mol­ecular arrangement of the title compound in the crystal. Dashed lines indicate the C—H⋯π inter­actions (ring centroids are shown as coloured spheres).

The mol­ecular structures and electronic properties of hexa­pyrrolic expanded porphyrins with different numbers of π-electrons and *meso*-like positions have been studied extensively (Sessler *et al.*, 1995 ▸; Saito & Osuka *et al.*, 2011 ▸; Setsune *et al.*, 2015 ▸). Furthermore, the crucial influence of a heteroatom on the macrocycle conformation of core-modified hexa­phyrins has been demonstrated (Narayanan *et al.*, 1998 ▸, 1999 ▸). [24]Amethyrin is a hexa­pyrrolic expanded porphyrin that has recently been a focus of theoretical studies, and exploring the mol­ecular structures and electronic properties of its derivatives is highly desirable. Di­thia­amethyrin is a core-modified a­methyl­ine with Group-16 heterocycles and *meso*-di­chloro­phenyl groups (Alka *et al.*, 2019 ▸). In a continuation of our research on the synthesis and characterization of expanded porphyrin derivatives (Ishimaru *et al.*, 2015 ▸, 2022 ▸), we synthesized the title compound, [24]di­thia­amethyrin(1.0.0.1.0.0), and elucidated its crystal structure. It has a highly planar macrocyclic core and its ^1^H NMR chemical shifts indicate strong anti­aromaticity. Its mol­ecular structure is shown in Fig. 1 ▸. Its planarity is reinforced by two intra­molecular N1—H1⋯N2 hydrogen bonds (Table 1 ▸) in the dipyrromethene moiety, as shown in Fig. 1 ▸. All the bond lengths are consistent with those reported in a previous study on other di­thia­amethyrins (Ishimaru *et al.*, 2015 ▸). The mean plane deviation (MPD) value of the 32 macrocyclic atoms is 0.0416 Å, and the *meso*-phenyl ring is twisted by 92.51° from the mean plane of [24]di­thia­amethyrin(1.0.0.1.0.0). Neighboring mol­ecules form dimers *via* inter­molecular C—H⋯Cl and C—H⋯π inter­actions owing to the H6⋯Cl1 (2.93 Å), H17*A*⋯Cl2 (3.34 Å), and C3—C11 (3.36 Å) distances, as shown in Fig. 2 ▸.

## Synthesis and crystallization

The title compound was prepared by a modified previously reported method by Ishimaru *et al.* (2015 ▸). 2,5-Bis­(4-propyl-2-pyrrol­yl)thio­phene (200 mg) was dissolved in CH_2_Cl_2_ (*ca* 300 mL) under an Ar atmosphere, to which 3,5-di­chloro­benzaldehyde (68.4 µL) and tri­fluoro­acetic acid (160 µL) were added. The reaction mixture was stirred for 3 h, *p*-chloranil (544 mg) was added to it, and the mixture was stirred overnight at ambient temperature. Then, the mixture was neutralized with an aqueous NaHCO_3_ solution, and the crude products were passed through an alumina column. Finally, the products were purified by chromatography on a silica gel column using chloro­form as elute. The third blue (2%) fraction afforded the title compound. The compound was recrystallized from a mixture of hexane and chloro­form. Purple plates of suitable quality for diffraction were obtained by slow-diffusing hexane into chloro­form. ^1^H NMR (400 MHz, CDCl_3_): δ (ppm) 24.0 (*br*, 2H, pyrrole NH), 7.20– 6.87 (*m*, 6H, *o, p*-Ph), 5.05 (*s*, 4H, thio­phene β-H), 4.61 (*s*, 4H, pyrrole β-H), 0.76 (*sextet*, 8H, CH_2_C**H**_2_CH_3_), 0.52 (*t*, 8H,C**H**_2_CH_2_CH_3_), 0.32 (*t*, 12H, CH_2_CH_2_C**H**_3_); MALDI–TOF MS found = 904.254, monoisotopic mass = 904.176 calculated for C_50_H_44_Cl_4_N_4_S_2_. UV–vis (CH_2_Cl_2_): λ = 387, 500 nm.

## Refinement

Crystal data, data collection, and structure refinement details are summarized in Table 2 ▸.

## Supplementary Material

Crystal structure: contains datablock(s) global, I. DOI: 10.1107/S2414314623007666/xu4052sup1.cif

Structure factors: contains datablock(s) I. DOI: 10.1107/S2414314623007666/xu4052Isup2.hkl

CCDC reference: 2292415

Additional supporting information:  crystallographic information; 3D view; checkCIF report

## Figures and Tables

**Table 1:** Hydrogen-bond geometry (Å, °) *Cg*1, *Cg*2 and *Cg*4 are the centroids of the S2/C1–C4, N1/C5–C8 and C20–C25, respectively.

*D*—H⋯*A*	*D*—H	H⋯*A*	*D*⋯*A*	*D*—H⋯*A*
N1—H1*N*⋯N2	0.86 (2)	1.92 (2)	2.6013 (19)	135.7 (18)
C14—H14*A*⋯*Cg*4	0.99	2.61	3.492 (2)	149
C14—H14*B*⋯*Cg*1^i^	0.99	2.83	3.522 (2)	128
C19—H19*C*⋯*Cg*2^ii^	0.99	2.90	3.713 (2)	141

Symmetry codes: (i) 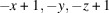 ; (ii) 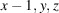 .

**Table 2:** Experimental details

Crystal data	
Chemical formula	C_50_H_44_Cl_4_N_4_S_2_
*M*_r_	906.81
Crystal system, space group	Triclinic, *P* 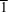
Temperature (K)	200
*a*, *b*, *c* (Å)	8.5016 (11), 10.1996 (16), 13.4724 (16)
α, β, γ (°)	104.368 (1), 91.416 (1), 99.030 (1)
*V* (Å^3^)	1115.2 (3)
*Z*	1
Radiation type	Mo *K*α
μ (mm^−1^)	0.40
Crystal size (mm)	0.12 × 0.04 × 0.02

Data collection	
Diffractometer	Bruker APEXII CCD area-detector
Absorption correction	Multi-scan (*SADABS*; Krause *et al.*, 2015 ▸)
No. of measured, independent and observed [*I* > 2σ(*I*)] reflections	11997, 4534, 3696
*R*_int_	0.025
(sin θ/λ)_max_ (Å^−1^)	0.625

Refinement	
*R*[*F* ^2^ > 2σ(*F* ^2^)], *wR*(*F* ^2^), *S*	0.034, 0.090, 1.22
No. of reflections	4534
No. of parameters	277
H-atom treatment	H atoms treated by a mixture of independent and constrained refinement
Δρ_max_, Δρ_min_ (e Å^−3^)	0.25, −0.32

Computer programs: *APEX2*, *SAINT*, *XPREP* and *XCIF* (Bruker 2014 ▸), *SHELXT2014* (Sheldrick, 2015*a* ▸), *SHELXL2014* (Sheldrick, 2015*b* ▸) and *ORTEP-3 for Windows* (Farrugia, 2012 ▸), .

## full crystallographic data

### Crystal data

**Table:**

C_50_H_44_Cl_4_N_4_S_2_	*Z* = 1
*M**_r_* = 906.81	*F*(000) = 472
Triclinic, *P*1	*D*_x_ = 1.350 Mg m^−^^3^
*a* = 8.5016 (11) Å	Mo *K*α radiation, λ = 0.71073 Å
*b* = 10.1996 (16) Å	Cell parameters from 3988 reflections
*c* = 13.4724 (16) Å	θ = 2.4–27.9°
α = 104.368 (1)°	µ = 0.40 mm^−^^1^
β = 91.416 (1)°	*T* = 200 K
γ = 99.030 (1)°	Needle, purple
*V* = 1115.2 (3) Å^3^	0.12 × 0.04 × 0.02 mm

### Data collection

**Table:**

Bruker APEXII CCD area-detector diffractometer	4534 independent reflections
Radiation source: Bruker TXS fine-focus rotating anode	3696 reflections with *I* > 2σ(*I*)
Bruker Helios multilayer confocal mirror monochromator	*R*_int_ = 0.025
Detector resolution: 8.333 pixels mm^-1^	θ_max_ = 26.4°, θ_min_ = 1.6°
φ and ω scans	*h* = −10→10
Absorption correction: multi-scan (SADABS; Krause *et al.*, 2015)	*k* = −12→12
	*l* = −16→16
11997 measured reflections	

### Refinement

**Table:**

Refinement on *F*^2^	0 restraints
Least-squares matrix: full	Hydrogen site location: mixed
*R*[*F*^2^ > 2σ(*F*^2^)] = 0.034	H atoms treated by a mixture of independent and constrained refinement
*wR*(*F*^2^) = 0.090	*w* = 1/[σ^2^(*F*_o_^2^) + (0.0409*P*)^2^] where *P* = (*F*_o_^2^ + 2*F*_c_^2^)/3
*S* = 1.22	(Δ/σ)_max_ < 0.001
4534 reflections	Δρ_max_ = 0.25 e Å^−^^3^
277 parameters	Δρ_min_ = −0.32 e Å^−^^3^

### Special details

**Table:**

Geometry. All esds (except the esd in the dihedral angle between two l.s. planes) are estimated using the full covariance matrix. The cell esds are taken into account individually in the estimation of esds in distances, angles and torsion angles; correlations between esds in cell parameters are only used when they are defined by crystal symmetry. An approximate (isotropic) treatment of cell esds is used for estimating esds involving l.s. planes. Least-squares planes (x,y,z in crystal coordinates) and deviations from them (* indicates atom used to define plane) 6.9622 (0.0034) x - 7.0043 (0.0050) y + 2.9660 (0.0089) z = 1.0352 (0.0023) * 0.0014 (0.0011) C20 * 0.0028 (0.0011) C21 * -0.0041 (0.0011) C22 * 0.0009 (0.0012) C23 * 0.0033 (0.0012) C24 * -0.0044 (0.0012) C25 Rms deviation of fitted atoms = 0.0031 - 3.3824 (0.0012) x - 3.1159 (0.0024) y + 12.3311 (0.0021) z = 2.9131 (0.0011) Angle to previous plane (with approximate esd) = 88.176 ( 0.038 ) * 0.0039 (0.0012) C1 * -0.0849 (0.0013) C2 * -0.0688 (0.0014) C3 * 0.0266 (0.0015) C4 * 0.0325 (0.0015) C5 * -0.0332 (0.0014) C6 * -0.0354 (0.0013) C7 * 0.0386 (0.0014) C8 * 0.0337 (0.0014) C9 * -0.0004 (0.0015) C10 * -0.0426 (0.0014) C11 * -0.0522 (0.0013) C12 * -0.0170 (0.0013) C13 * 0.1059 (0.0007) S1 * 0.0882 (0.0014) N1 * 0.0051 (0.0013) N2 3.1186 (0.0019) C1_$1 3.2074 (0.0020) C2_$1 3.1912 (0.0021) C3_$1 3.0959 (0.0021) C4_$1 3.0900 (0.0021) C5_$1 3.1557 (0.0021) C6_$1 3.1579 (0.0020) C7_$1 3.0839 (0.0020) C8_$1 3.0887 (0.0020) C9_$1 3.1228 (0.0021) C10_$1 3.1650 (0.0020) C11_$1 3.1746 (0.0020) C12_$1 3.1394 (0.0020) C13_$1 3.0165 (0.0016) S1_$1 3.0342 (0.0020) N1_$1 3.1173 (0.0020) N2_$1 Rms deviation of fitted atoms = 0.0516 average 3.12 (5)
Refinement. The N-bound H atom was located in a difference Fourier map and freely refined, whereas the C-bound H atoms were included in calculated positions and treated as riding atoms: C—H = 0.95-0.99Å with *U*_iso_(H) = 1.5U_eq_(C) for methyl H atoms and = 1.2U_eq_(C) for aromatic H atoms.

### Fractional atomic coordinates and isotropic or equivalent isotropic displacement parameters (Å^2^)

**Table:**

	*x*	*y*	*z*	*U*_iso_*/*U*_eq_	
C1	0.83198 (19)	0.42761 (16)	0.57282 (12)	0.0240 (3)	
C2	0.89838 (19)	0.31039 (15)	0.55421 (12)	0.0257 (4)	
H2	1.0042	0.3083	0.5774	0.031*	
C3	0.79371 (19)	0.19374 (16)	0.49733 (12)	0.0267 (4)	
H3	0.8216	0.1047	0.4784	0.032*	
C4	0.64726 (19)	0.22118 (15)	0.47183 (12)	0.0242 (4)	
C5	0.51325 (19)	0.12833 (15)	0.41208 (12)	0.0245 (3)	
C6	0.49544 (19)	−0.01215 (16)	0.36638 (12)	0.0261 (4)	
H6	0.5728	−0.0689	0.3702	0.031*	
C7	0.34564 (19)	−0.05549 (15)	0.31416 (12)	0.0249 (4)	
C8	0.27007 (19)	0.06199 (15)	0.32911 (12)	0.0239 (3)	
C9	0.11941 (19)	0.08440 (15)	0.29306 (12)	0.0235 (3)	
C10	0.07289 (19)	0.21206 (15)	0.30979 (12)	0.0242 (3)	
C11	−0.07339 (19)	0.25311 (16)	0.27662 (12)	0.0241 (3)	
C12	−0.05427 (19)	0.39144 (16)	0.31603 (12)	0.0264 (4)	
H12	−0.1287	0.4491	0.3081	0.032*	
C13	0.09840 (19)	0.43419 (15)	0.37157 (12)	0.0239 (3)	
C14	0.2943 (2)	−0.20088 (15)	0.24958 (12)	0.0268 (4)	
H14A	0.1762	−0.2215	0.2415	0.032*	
H14B	0.3309	−0.2651	0.2859	0.032*	
C15	0.3603 (2)	−0.22472 (17)	0.14366 (13)	0.0350 (4)	
H15A	0.3158	−0.1666	0.1049	0.042*	
H15B	0.4777	−0.1962	0.1516	0.042*	
C16	0.3206 (3)	−0.3744 (2)	0.08244 (16)	0.0564 (6)	
H16A	0.3605	−0.4328	0.1217	0.085*	
H16B	0.3710	−0.3856	0.0169	0.085*	
H16C	0.2046	−0.4010	0.0694	0.085*	
C17	−0.21765 (19)	0.16697 (16)	0.21235 (13)	0.0277 (4)	
H17A	−0.2672	0.0985	0.2480	0.033*	
H17B	−0.1832	0.1163	0.1461	0.033*	
C18	−0.3418 (2)	0.25029 (18)	0.19110 (14)	0.0345 (4)	
H18A	−0.2922	0.3195	0.1561	0.041*	
H18B	−0.3777	0.2999	0.2572	0.041*	
C19	−0.4859 (2)	0.1619 (2)	0.12488 (16)	0.0460 (5)	
H19A	−0.4519	0.1158	0.0581	0.069*	
H19B	−0.5634	0.2200	0.1149	0.069*	
H19C	−0.5354	0.0929	0.1590	0.069*	
C20	0.00756 (19)	−0.04103 (15)	0.23484 (12)	0.0238 (3)	
C21	−0.09188 (19)	−0.11751 (15)	0.28815 (13)	0.0254 (4)	
H21	−0.0922	−0.0879	0.3608	0.031*	
C22	−0.18983 (19)	−0.23646 (16)	0.23485 (13)	0.0268 (4)	
C23	−0.1916 (2)	−0.28359 (16)	0.12933 (13)	0.0314 (4)	
H23	−0.2590	−0.3660	0.0935	0.038*	
C24	−0.0920 (2)	−0.20670 (17)	0.07785 (13)	0.0315 (4)	
C25	0.00693 (19)	−0.08578 (16)	0.12869 (12)	0.0275 (4)	
H25	0.0735	−0.0342	0.0913	0.033*	
Cl1	−0.31419 (5)	−0.33119 (4)	0.30225 (4)	0.03638 (13)	
Cl2	−0.08880 (7)	−0.26370 (5)	−0.05468 (4)	0.05066 (16)	
H1N	0.353 (2)	0.252 (2)	0.4028 (15)	0.050 (6)*	
N1	0.37688 (16)	0.17056 (14)	0.38987 (11)	0.0262 (3)	
N2	0.17494 (15)	0.32997 (13)	0.36802 (10)	0.0255 (3)	
S1	0.63866 (5)	0.39334 (4)	0.51941 (3)	0.02906 (12)	

### Atomic displacement parameters (Å^2^)

**Table:**

	*U*^11^	*U*^22^	*U*^33^	*U*^12^	*U*^13^	*U*^23^
C1	0.0230 (8)	0.0203 (8)	0.0263 (8)	0.0017 (6)	−0.0030 (7)	0.0032 (7)
C2	0.0219 (8)	0.0245 (8)	0.0294 (9)	0.0041 (7)	−0.0026 (7)	0.0048 (7)
C3	0.0286 (9)	0.0178 (8)	0.0317 (9)	0.0059 (7)	0.0004 (7)	0.0016 (7)
C4	0.0271 (9)	0.0162 (7)	0.0269 (8)	0.0024 (6)	0.0004 (7)	0.0017 (6)
C5	0.0242 (8)	0.0199 (8)	0.0275 (8)	0.0023 (6)	−0.0003 (7)	0.0033 (7)
C6	0.0257 (9)	0.0198 (8)	0.0308 (9)	0.0039 (6)	−0.0004 (7)	0.0029 (7)
C7	0.0275 (9)	0.0186 (8)	0.0262 (8)	0.0004 (7)	0.0027 (7)	0.0032 (7)
C8	0.0234 (8)	0.0175 (8)	0.0261 (8)	−0.0014 (6)	−0.0014 (7)	0.0005 (6)
C9	0.0237 (8)	0.0204 (8)	0.0229 (8)	−0.0004 (6)	0.0012 (7)	0.0019 (6)
C10	0.0247 (8)	0.0184 (8)	0.0261 (8)	−0.0009 (6)	−0.0011 (7)	0.0025 (6)
C11	0.0235 (8)	0.0222 (8)	0.0242 (8)	0.0001 (6)	−0.0007 (7)	0.0038 (7)
C12	0.0258 (9)	0.0232 (8)	0.0299 (9)	0.0045 (7)	−0.0022 (7)	0.0060 (7)
C13	0.0252 (8)	0.0196 (8)	0.0251 (8)	0.0016 (6)	−0.0008 (7)	0.0038 (7)
C14	0.0273 (9)	0.0153 (8)	0.0342 (9)	0.0012 (6)	−0.0025 (7)	0.0013 (7)
C15	0.0388 (11)	0.0252 (9)	0.0362 (10)	0.0046 (8)	0.0025 (8)	−0.0003 (8)
C16	0.0759 (16)	0.0315 (11)	0.0489 (13)	0.0056 (11)	0.0029 (12)	−0.0112 (10)
C17	0.0260 (9)	0.0231 (8)	0.0298 (9)	−0.0009 (7)	−0.0028 (7)	0.0025 (7)
C18	0.0290 (10)	0.0303 (9)	0.0412 (10)	0.0005 (7)	−0.0092 (8)	0.0075 (8)
C19	0.0346 (11)	0.0455 (12)	0.0538 (13)	−0.0041 (9)	−0.0158 (9)	0.0136 (10)
C20	0.0215 (8)	0.0169 (7)	0.0303 (9)	0.0028 (6)	−0.0014 (7)	0.0014 (7)
C21	0.0244 (9)	0.0207 (8)	0.0295 (9)	0.0048 (6)	−0.0008 (7)	0.0030 (7)
C22	0.0205 (8)	0.0211 (8)	0.0386 (10)	0.0029 (6)	0.0031 (7)	0.0076 (7)
C23	0.0276 (9)	0.0221 (8)	0.0376 (10)	−0.0033 (7)	−0.0056 (8)	−0.0001 (7)
C24	0.0346 (10)	0.0262 (9)	0.0282 (9)	0.0016 (7)	−0.0028 (8)	−0.0003 (7)
C25	0.0271 (9)	0.0224 (8)	0.0302 (9)	−0.0005 (7)	−0.0003 (7)	0.0044 (7)
Cl1	0.0293 (2)	0.0266 (2)	0.0536 (3)	−0.00007 (17)	0.0072 (2)	0.0134 (2)
Cl2	0.0637 (4)	0.0443 (3)	0.0294 (3)	−0.0123 (2)	−0.0027 (2)	−0.0038 (2)
N1	0.0247 (7)	0.0163 (7)	0.0335 (8)	0.0024 (6)	−0.0048 (6)	0.0000 (6)
N2	0.0252 (7)	0.0168 (6)	0.0306 (7)	0.0004 (5)	−0.0027 (6)	0.0012 (6)
S1	0.0236 (2)	0.0174 (2)	0.0414 (3)	0.00337 (16)	−0.00734 (18)	−0.00045 (17)

### Geometric parameters (Å, º)

**Table:**

C1—C2	1.371 (2)	C14—H14B	0.9900
C1—C13^i^	1.446 (2)	C15—C16	1.524 (2)
C1—S1	1.7256 (16)	C15—H15A	0.9900
C2—C3	1.406 (2)	C15—H15B	0.9900
C2—H2	0.9500	C16—H16A	0.9800
C3—C4	1.372 (2)	C16—H16B	0.9800
C3—H3	0.9500	C16—H16C	0.9800
C4—C5	1.442 (2)	C17—C18	1.519 (2)
C4—S1	1.7274 (15)	C17—H17A	0.9900
C5—N1	1.350 (2)	C17—H17B	0.9900
C5—C6	1.394 (2)	C18—C19	1.520 (2)
C6—C7	1.392 (2)	C18—H18A	0.9900
C6—H6	0.9500	C18—H18B	0.9900
C7—C8	1.420 (2)	C19—H19A	0.9800
C7—C14	1.511 (2)	C19—H19B	0.9800
C8—N1	1.3852 (19)	C19—H19C	0.9800
C8—C9	1.430 (2)	C20—C25	1.389 (2)
C9—C10	1.387 (2)	C20—C21	1.393 (2)
C9—C20	1.496 (2)	C21—C22	1.379 (2)
C10—N2	1.4088 (18)	C21—H21	0.9500
C10—C11	1.466 (2)	C22—C23	1.382 (2)
C11—C12	1.360 (2)	C22—Cl1	1.7431 (16)
C11—C17	1.502 (2)	C23—C24	1.380 (2)
C12—C13	1.431 (2)	C23—H23	0.9500
C12—H12	0.9500	C24—C25	1.387 (2)
C13—N2	1.323 (2)	C24—Cl2	1.7375 (18)
C13—C1^i^	1.446 (2)	C25—H25	0.9500
C14—C15	1.523 (2)	N1—H1N	0.86 (2)
C14—H14A	0.9900		
			
C2—C1—C13^i^	129.06 (15)	C14—C15—H15B	109.1
C2—C1—S1	110.85 (12)	H15A—C15—H15B	107.8
C13^i^—C1—S1	120.09 (12)	C15—C16—H16A	109.5
C1—C2—C3	113.04 (15)	C15—C16—H16B	109.5
C1—C2—H2	123.5	H16A—C16—H16B	109.5
C3—C2—H2	123.5	C15—C16—H16C	109.5
C4—C3—C2	113.38 (14)	H16A—C16—H16C	109.5
C4—C3—H3	123.3	H16B—C16—H16C	109.5
C2—C3—H3	123.3	C11—C17—C18	113.31 (13)
C3—C4—C5	128.64 (14)	C11—C17—H17A	108.9
C3—C4—S1	110.58 (12)	C18—C17—H17A	108.9
C5—C4—S1	120.77 (12)	C11—C17—H17B	108.9
N1—C5—C6	107.54 (14)	C18—C17—H17B	108.9
N1—C5—C4	122.37 (14)	H17A—C17—H17B	107.7
C6—C5—C4	130.08 (15)	C17—C18—C19	112.65 (15)
C7—C6—C5	108.72 (14)	C17—C18—H18A	109.1
C7—C6—H6	125.6	C19—C18—H18A	109.1
C5—C6—H6	125.6	C17—C18—H18B	109.1
C6—C7—C8	106.71 (13)	C19—C18—H18B	109.1
C6—C7—C14	121.77 (14)	H18A—C18—H18B	107.8
C8—C7—C14	131.34 (15)	C18—C19—H19A	109.5
N1—C8—C7	106.51 (14)	C18—C19—H19B	109.5
N1—C8—C9	120.11 (14)	H19A—C19—H19B	109.5
C7—C8—C9	133.36 (14)	C18—C19—H19C	109.5
C10—C9—C8	124.29 (14)	H19A—C19—H19C	109.5
C10—C9—C20	119.73 (14)	H19B—C19—H19C	109.5
C8—C9—C20	115.98 (13)	C25—C20—C21	119.52 (14)
C9—C10—N2	120.08 (14)	C25—C20—C9	120.83 (14)
C9—C10—C11	131.21 (14)	C21—C20—C9	119.58 (14)
N2—C10—C11	108.71 (13)	C22—C21—C20	119.60 (15)
C12—C11—C10	105.56 (14)	C22—C21—H21	120.2
C12—C11—C17	124.79 (15)	C20—C21—H21	120.2
C10—C11—C17	129.65 (14)	C21—C22—C23	121.91 (15)
C11—C12—C13	107.51 (14)	C21—C22—Cl1	119.24 (13)
C11—C12—H12	126.2	C23—C22—Cl1	118.85 (12)
C13—C12—H12	126.2	C24—C23—C22	117.68 (15)
N2—C13—C12	112.01 (14)	C24—C23—H23	121.2
N2—C13—C1^i^	121.23 (14)	C22—C23—H23	121.2
C12—C13—C1^i^	126.76 (15)	C23—C24—C25	122.05 (16)
C7—C14—C15	112.74 (13)	C23—C24—Cl2	119.06 (13)
C7—C14—H14A	109.0	C25—C24—Cl2	118.88 (13)
C15—C14—H14A	109.0	C24—C25—C20	119.23 (15)
C7—C14—H14B	109.0	C24—C25—H25	120.4
C15—C14—H14B	109.0	C20—C25—H25	120.4
H14A—C14—H14B	107.8	C5—N1—C8	110.52 (13)
C16—C15—C14	112.51 (15)	C5—N1—H1N	130.5 (14)
C16—C15—H15A	109.1	C8—N1—H1N	118.7 (14)
C14—C15—H15A	109.1	C13—N2—C10	106.20 (13)
C16—C15—H15B	109.1	C1—S1—C4	92.15 (8)
			
C13^i^—C1—C2—C3	179.71 (16)	C8—C7—C14—C15	94.1 (2)
S1—C1—C2—C3	−0.08 (18)	C7—C14—C15—C16	174.90 (16)
C1—C2—C3—C4	0.3 (2)	C12—C11—C17—C18	2.1 (2)
C2—C3—C4—C5	178.12 (15)	C10—C11—C17—C18	−177.42 (16)
C2—C3—C4—S1	−0.32 (18)	C11—C17—C18—C19	179.25 (15)
C3—C4—C5—N1	−177.74 (16)	C10—C9—C20—C25	89.16 (19)
S1—C4—C5—N1	0.6 (2)	C8—C9—C20—C25	−91.51 (19)
C3—C4—C5—C6	1.2 (3)	C10—C9—C20—C21	−93.89 (19)
S1—C4—C5—C6	179.48 (14)	C8—C9—C20—C21	85.44 (18)
N1—C5—C6—C7	0.81 (19)	C25—C20—C21—C22	−0.1 (2)
C4—C5—C6—C7	−178.24 (16)	C9—C20—C21—C22	−177.12 (14)
C5—C6—C7—C8	−0.32 (18)	C20—C21—C22—C23	0.7 (2)
C5—C6—C7—C14	175.29 (14)	C20—C21—C22—Cl1	−179.84 (12)
C6—C7—C8—N1	−0.27 (18)	C21—C22—C23—C24	−0.5 (3)
C14—C7—C8—N1	−175.30 (16)	Cl1—C22—C23—C24	−179.97 (13)
C6—C7—C8—C9	177.90 (17)	C22—C23—C24—C25	−0.3 (3)
C14—C7—C8—C9	2.9 (3)	C22—C23—C24—Cl2	179.18 (13)
N1—C8—C9—C10	3.8 (2)	C23—C24—C25—C20	0.8 (3)
C7—C8—C9—C10	−174.14 (17)	Cl2—C24—C25—C20	−178.67 (13)
N1—C8—C9—C20	−175.46 (14)	C21—C20—C25—C24	−0.6 (2)
C7—C8—C9—C20	6.6 (3)	C9—C20—C25—C24	176.39 (15)
C8—C9—C10—N2	−1.8 (2)	C6—C5—N1—C8	−1.00 (18)
C20—C9—C10—N2	177.47 (13)	C4—C5—N1—C8	178.14 (14)
C8—C9—C10—C11	178.31 (16)	C7—C8—N1—C5	0.79 (18)
C20—C9—C10—C11	−2.4 (3)	C9—C8—N1—C5	−177.67 (14)
C9—C10—C11—C12	179.53 (17)	C12—C13—N2—C10	−0.63 (18)
N2—C10—C11—C12	−0.36 (17)	C1^i^—C13—N2—C10	179.02 (14)
C9—C10—C11—C17	−0.9 (3)	C9—C10—N2—C13	−179.30 (14)
N2—C10—C11—C17	179.25 (15)	C11—C10—N2—C13	0.61 (17)
C10—C11—C12—C13	−0.01 (17)	C2—C1—S1—C4	−0.08 (13)
C17—C11—C12—C13	−179.65 (15)	C13^i^—C1—S1—C4	−179.90 (13)
C11—C12—C13—N2	0.41 (19)	C3—C4—S1—C1	0.23 (13)
C11—C12—C13—C1^i^	−179.21 (15)	C5—C4—S1—C1	−178.35 (13)
C6—C7—C14—C15	−80.34 (19)		

Symmetry code: (i) −*x*+1, −*y*+1, −*z*+1.

### Hydrogen-bond geometry (Å, º)

*Cg*1, *Cg*2 and *Cg*4 are the centroids of the S2/C1–C4, N1/C5–C8 and C20–C25 rings, respectively.

**Table:**

*D*—H···*A*	*D*—H	H···*A*	*D*···*A*	*D*—H···*A*
N1—H1*N*···N2	0.86 (2)	1.92 (2)	2.6013 (19)	135.7 (18)
C14—H14*A*···*Cg*4	0.99	2.61	3.492 (2)	149
C14—H14*B*···*Cg*1^ii^	0.99	2.83	3.522 (2)	128
C19—H19*C*···*Cg*2^iii^	0.99	2.90	3.713 (2)	141

Symmetry codes: (ii) −*x*+1, −*y*, −*z*+1; (iii) *x*−1, *y*, *z*.
